# Supplementary material for: Qualitative Research Studies Addressing Patient-Practitioner Communication about Online Health Information
Source: Int J Environ Res Public Health. 2022 Oct 27;19(21):14004. doi: 10.3390/ijerph192114004 (PMC9659101; doi:10.3390/ijerph192114004)
Supplement: Supplementary file 1 [file ijerph-19-14004-s001.zip › ijerph-1920152-supplementary.pdf]

Article	Participants and methods	Main findings
		**Patients perspective**
**Diviani et al. 2019. Norway, Australia, Israel, Netherlands, Switzerland**	165 participants from 5 countries (OHI seeking)<br><br>Semi-structured in-depth interviews. Transcribed verbatim analyzed with thematic analysis	**Seeking and finding OHI:** OHI was used to self-diagnose, or avoid the GP. Sometimes performed in conjunction with the GP: to prepare or complement information, sometimes challenge it. Searching for others. Makes information easily accessible. Google as a starting point, but many used specific reginal sites (e.g helsenorge.no).<br>**Understanding OHI:** Medical jargon could result in less searching. Difficult to know what information to trust, how they evaluate websites.<br>**Applying information**: Active application of knowledge: modifying health behavior based on OHI (diet or work-out related). No one change their medication without discussing with their practitioner. there are some reluctances towards discussing online information with the doctor
**Fahmer, et al 2021. Germany**	Patients with breast or gynecological cancer diagnosis in acute care (19), Undergoing rehabilitation (20) attending self-help groups(16)<br><br>Semi structured interviews, convenience sampling. Transcribed verbatim. Analyzed in ATLAS.ti 8.2<br>qualitative content analysis.	**Challenges:** Gain information according to own needs. Barriers: doctor patient communication, situationally limited information processing, Difficult access to information. Internet as source of information. Information evaluation and treatment decision.<br>**Personal competencies:** Self-regulation of information needs. Self-directed information acquisition – media and social competencies. Communication skills in the doctor patient conversation, Internet competencies. Self-directed decisions according to own needs. Characteristics: Medical, disease-related previous knowledge, interest, self-efficacy, self-concept (active role). Trust in the doctor.<br>**Helpful support:** relatives and friends conversations, support during consultations.<br>Professionals needs-oriented information, Patient centered communication. Counselling center, hospital, rehab, and interdisciplinary cooperation.
**Heldal, et al (2020) Norway**	17 Senior citizens from Norway<br><br>Open ended interviews, analyzed through the lens of predetermined categories,	Due to fast-speed development of digital tools even those previously profound data users were lagging. They often asked younger family members for help. They searches were motivated by concerns or wishes to help others, or sometimes curiosity.<br>Online Information was often discussed with peers, especially regarding weather the information indicated that a GP should be contacted or not. The article also reported a difference between the study by Divianis younger population and their own senior population group in terms of confidence when searching and applying OHI.

**Lee, et al, 2014 Australia**	17 adult chronic disease patient who used the internet to find health information<br><br>Interview study, audio recorded and transcribed verbatim. Analysis was conducted in NVivo 10. Two approaches to thematic analyses where used: Datadriven – codes inductively generated from the data, and Theory driven approach, whereby a framework of codes was developed from theory of anticipated results	Participants commonly searched Health information related information to understand their medical condition and medications prescribed. They did this to be more informed about their health, and it was commonly related to a consultation with a HCP and often performed before and/or after. To make decisions weather to seek medical attention, or to make decisions about recommendations made by their HCP. Patients also mostly searched information relevant to their own condition.<br>Barriers to access information were reported as external factors: Availability/accessibility of content (e.g. research journals), use of medical jargon, inconsistency and volume of information, poor relationship with health professional (low trust). Internal factors: Limited eHL, lack of knowledge of credible websites, unsure of information need, limited time, lack of motivation. Needs there were reported to make use of OHI was: Feedback. greater availability, designed destinations for credible information, HCP input and advice on good information sources. Trusting their information sources, and their HCP was also important factors.
**Longo, et al. 2010 USA**	46 participants in 9 focus groups. (Patients with DIA2)<br><br>Focus groups. Interviews guided by (McNamera) and Health information model. Transcripts were coded to identify active information seeking and passive information seeking. Identifying themes through collaborative process	Media (Tv and newspapers) played an inadvertent role in patient passively receiving information. Searching the internet was part of active information seeking. Other important sources were print media, verbal and written information from HCP, and Relatives and friends. HCP: information overload could be a hindrance. Diabetes related online information - patient brought what they found to their HCP for confirmation – this was seen as a critical function for the HCP during the encounters. HCP were the most frequent use of information source in general. HL makes a difference: patients across various educational levels fronted that some information could be difficult to enhance.<br>Some also searched information to challenge the GP if they did not agree with treatment plans or suggestions.
**Magsamaen-Conrad et al (2019) USA**	91 participants (55 men and 36 women), convenience sapling.<br><br>Semi-structured, in-depth interviews Inductive analysis.	The categories that emerged in the analysis were 1) Seeking inline information, 2) Outside influences, and 3) Health co-management.<br>1) Many used online information as supplemental to the doctor's information both before and after a medical visit, - However, it was seen as supplemental information, not information to be used instead of a medical visit. The professional was still prioritized. Online information was often used to reduce uncertainty and save face.<br>2) Family members attitudes towards the participants digital literacy could influence their usage of various tools. As if they were slow, or just not seen as internet

		users, family members could exclude them form discussions, or be impatient when looking things up. Many women described how they also looked up various stuff online to co-manage their partners health issues
**Neill et al, 2014 United Kingdom**	27 parents (24 mothers and 3 fathers) of children under 5 years old presenting with acute illness from east midlands in the UK, and in south Asian and gypsy/travelling communities<br><br>Exploratory qualitative study. Focus group discussions and interviews. Grounded theory /thematic analysis. Coded in NVivo 10. Constant comparative analysis	Internet was the default information source. It was searched when they had a diagnosis, when they were fairly certain it is nothing, but just want to check, and when they wanted to check delf-diagnosis to be sure whether they were to contact the doctor or not. There were various ways of contacting the health care centers, and the parents had various needs when making the contact. From diagnosis, reassurance, information on what to do and when to worry and help to understand online information. Referrals to websites were seen as positive (however, rarely used when the child suffered another episode of acute illness). It was reported that HCP altered their responses in accordance with how they perceived the parents' competencies (especially regarding first VS more children). The attitudes of the HCP and available time also made a difference in access to information. So did Power imbalance. Other reported influential factors were low literacy, timing of information, gender (dads, got no hard copy), Internet: How the parents judged the quality of the web page.
**Protheroe, 2012, United Kingdom**	Chronic disease patients, 35 participants<br><br>Semi-structured interviews, Framework approach	Looking at variations between groups of different socioeconomic statis (SES). Active information seeking (people with high SES in general sought more information than people with low SES. Both groups were reluctant to internet, due to information overload and lack of quality insurance of the information). Using knowledge and understanding (using health knowledge in decision making and active self-management was linked to narratives of active information seeking). The impact of relationships with the healthcare system and professionals (The patient "role" was deemed as supposed to be passive (paternalistic view on GP) these expectations were held by most low SES participants while High SES perceived more responsibility in looking up information and taking action themselves)
**Silver, 2015, Canada**	Citizens above 50 years. 56 participants (30 women, 26 men) face-face interviews. (NVivo, for transcripts, Stata to for demographic information. Grounded theory (Strauss, 1990) - deductive analysis (Mayring,2004), and thematic analysis (Braun and Clarke, 2006)	Participants who discussed their internet search with doctor were more concerned about credibility or limitations of the online information, or limitations in their own ability to search and evaluate the information. Patients who did not discuss with their doctors were more concerned about non-physical harm that could be done, and concerns related to anxiety. Barriers to communicate with the doctor: (worries about what the doctors would think of you, ideas of what the doctor wants to hear/like to hear about, some goes to the internet instead of the doctor, and sometimes it just did not "come up") Facilitators: (Family members who helped them

		remember, doctor-initiated inquiries, Advertisements that encouraged participants to talk to the doctor)
**Vamos et al, 2019, USA**	17 pregnant women Focus group discussions	**Access** (to information): HCP, Search engines and internet sites, other HCP-related sources, friends and family, mobile applications, books.<br>**Understand:** through conversations with others, pictures and visuals, Numbers and statistics, Tailored information<br>**Appraise (**how they evaluated information). Credibility of the HCP, presence of advertisements, having multiple sources of information, personal health circumstances, values, and gestational age.<br>**Apply**<br>Participants usen information to inform communication with HCP and to act on and make health related decisions
	**Practitioners and Patients**	
**Jiang, et al. 2021**<br><br>**China**	29 patients with >COPD (from three different follow-up programs) and 23 HCP's working with CHPD rehabilitation. (Nurses, practitioners and physiotherapists)<br><br>Semi structured interviews. Purposive sampling. Data was transcribed verbatim and analyzed with inductive thematic analysis method.	Elderly populations and online information:<br>They search for lack of information or for wonder medicines?<br>Some were reluctant to search due to psychological discomfort (like finding out that COPD could not be cured).<br>Some apply the information well; others miss interpret or read wrongful information that interferes with treatment (e.g. finding that some says smoking could be good).<br>Some patient reported being overwhelmed by the amount of information difficult to distinguish what is good and what is bad. - Lack of basic levels of competencies (illiterate, memory loss, slower learning abilities). - Patient confidence in the meeting with digital solutions. - Being too lazy<br>It's their responsibility for your own health VS time management (too busy with grandkids)<br>Forwarding online information: some do it to help others, others are afraid they will send wrongful information.<br>Find it novel (interesting?) to search online<br>Superior to others if you could search and apply online information.<br>Distance to the health care practitioners. - "smart devices are not people"<br>Accessing health information online: social prejudice due to fraud, privacy, and incorrect information.<br>How HCP talked about online information mattered, Patients were also more likely to follow the advice of trusted HCPs regarding online information.
**Hart, et al (2004) United Kingdom**	47 patients (32 women and 15 men) who were in contact with HC for hormone replacement therapy.<br><br>10 hcp (4 consultant doctors, 3 GP's 2 specialist nurses and 1 psychologist	Lack of awareness about where they find the information. Little interest in validity of sources. practitioners were more worried about their own online skills. No patients were given information about online information sources from their practitioner. (No online information observed during observations). Patients would rather use trusted HCP than internet. "The power of the internet" even those not using it, felt they should. HCP: reported worries that internet would encourage patients to challenge the "medical authority"

	Semi structured interviews, (21 participants were interviewed twice) transcribed verbatim. Data was analyzed in NVivo.<br><br>16 consultations, were observed	*This article is written in the early WWW age. And the internet was fairly less accessible at this point. Interesting difference of the findings compared to newer studies.*
	**Practitioners**	
**Caiata-Zufferey, 2012 Switzerland**	17 Italian-Swizz physicians (from various specialist fields)<br><br>Qualitative semi structured interviews. Analysis: Grounded theory (Strauss and Corbin 1990) - constant comparative method	Four communicative strategies for managing the internet informed patient: 1. Resistance to online information (Neutralize the IIP) 2. Repairing online information (Correct IIP and relate them to the doctor's point of view) 3. Construction around inline information (build a shared reality using the online information as a springboard) 4. enhancement of online information (empower IIP to by providing the instruments to obtain quality information. Physicians' behavior and choice of strategy did relate to the patients perceived level of Health literacy.
**Fredriksen, 2018, Norway**	13 (4 midwives, 4 physiotherapists, 5 general practitioners)<br><br>Thematic, semi structured interviews. Analysis and sampling done in a parallel process. Thematic analysis	visibility of eHL in the consultations (Knowledge, arriving from somewhere, google? pre diagnosis? - some HCP ask about the knowledge level. EHL= elephant in the room) Challenge of incorporating eHL into consultation. - ambivalence to internet information. depending on how it is used (some predetermine their treatment and diagnosis, while otters use it to understand and gain knowledge - this affects the consultation)<br>The role of the practitioner challenged?
**Sjøstrøm 2019, Sweden**	9 Primary healthcare nurses<br><br>Qualitative research, Semi-structured interviews. Qualitative content analysis (Graneim and Lundman)	Internet informed patients change the rules of the game - facing downsides of googling (Confusion due to contradiction, Disputes related to differing opinions, Unfolded anxiety among patients) Patients as main actors (Patient as lay experts, self-care initiatives facilitated, Patients as equal partners) Nurse role challenged; (Being considered unnecessary, Keeping updated is essential, coaching instead of controlling,
**Woodward-Kron, 2014, Australia and Switzerland**	21 physicians Semi-structured interviews.<br><br>interactive process, inductive analysis	Conceptual model on Interaction with internet-based health information - physician - patient benefits? Physicians engaging their patients in internet information, (variated from type of physician, also on type of patent and their families, as well as time in the consultation the setting of the consultation. and communication activities. The online

	information: Physicians alert to variable quality in online information and what that does to their patients
